# Maternal depression during pregnancy and offspring depression in adulthood: role of child maltreatment

**DOI:** 10.1192/bjp.bp.114.156620

**Published:** 2015-09

**Authors:** Dominic T. Plant, Carmine M. Pariante, Deborah Sharp, Susan Pawlby

**Affiliations:** **Dominic T. Plant**, PhD, Department of Psychological Medicine, Institute of Psychiatry, Psychology & Neuroscience, King's College London, UK; **Carmine M. Pariante**, MD, FRCPsych, PhD, Department of Psychological Medicine, Institute of Psychiatry, Psychology & Neuroscience, King's College London, UK; **Deborah Sharp**, MA, FRCGP, PhD, School of Social and Community Medicine, University of Bristol, UK; **Susan Pawlby**, MA, PhD, CPsychol, Department of Psychological Medicine, Institute of Psychiatry, Psychology & Neuroscience, King's College London, UK

## Abstract

**Background**

Studies have shown that maternal depression during pregnancy predicts offspring depression in adolescence. Child maltreatment is also a risk factor for depression.

**Aims**

To investigate (a) whether there is an association between offspring exposure to maternal depression in pregnancy and depression in early adulthood, and (b) whether offspring child maltreatment mediates this association.

**Method**

Prospectively collected data on maternal clinical depression in pregnancy, offspring child maltreatment and offspring adulthood (18–25 years) DSM-IV depression were analysed in 103 mother–offspring dyads of the South London Child Development Study.

**Results**

Adult offspring exposed to maternal depression in pregnancy were 3.4 times more likely to have a DSM-IV depressive disorder, and 2.4 times more likely to have experienced child maltreatment, compared with non-exposed offspring. Path analysis revealed that offspring experience of child maltreatment mediated the association between exposure to maternal depression in pregnancy and depression in adulthood.

**Conclusions**

Maternal depression in pregnancy is a key vulnerability factor for offspring depression in early adulthood.

Research has documented a link between maternal postnatal depression and offspring depression.^1–3^ Research has focused less on the impact of depression during pregnancy on offspring depressive psychopathology. Nevertheless, a handful of longitudinal studies have demonstrated an association between maternal depression in pregnancy and the development of depressive psychopathology in exposed offspring, but the evidence so far is limited to adolescence, that is, from 13 through to 18 years of age.^4–6^ To our knowledge, no prospective study has examined the association between offspring exposure to maternal clinical depression *in utero* and depression in adulthood; moreover, the pathways underpinning this association have not yet been clarified. Indeed, most research to date has focused on the broader relationship between maternal prenatal stress and offspring neurodevelopmental and health outcomes in later life, such as stress physiology, brain plasticity, immunity and chronic metabolic diseases, in addition to psychopathology.^7–10^

Evidence has also shown that maternal affective psychopathology in pregnancy is associated with offspring vulnerability to adverse childhood experiences.^11,12^ Specifically, in a recent study, maternal depression and anxiety in pregnancy was found to predict offspring victimisation in middle childhood, whereby there was a significant direct effect, as well as significant indirect effects through parental maladaptive parenting and conflict.^12^ Moreover, we have previously reported a link between a mother's experience of depression in pregnancy and her offspring's experience of harsh parental discipline and sexual and physical abuse at 11 years.^11^ Notably, in these particular instances, mothers were not the main perpetrators of violence, and were never involved in any instances of sexual abuse. Rather, peers and other adults in the home were primary perpetrators. Offspring of prenatally depressed and anxious mothers also exhibit more emotional reactivity and difficult temperament.^13–17^ Collectively, these data suggest that the link between maternal affective disorders in pregnancy and offspring maltreatment is likely underpinned by changes to mothers' caregiving and attachment behaviours and her ability to protect her child, the impact of aggregated domestic risks, as well as potential foetal programming of a more emotionally labile offspring temperament, all of which could increase vulnerability to being maltreated.^12,18,19^

Research has also identified a robust association between experience of child maltreatment and the later development of depression.^20–25^ Indeed, a range of types of abuse and neglect are associated with an increased risk for depression in adulthood.^20,22^ Some studies have reported a ‘dose–response’ relationship between severity of maltreatment and depression, as well as a link with depression that is more difficult to treat and has a chronic course.^21,23,25^ Preclinical studies in rodents and non-human primates have also shown that prolonged separation during the early sensitive period of development leads to behavioural changes in the offspring that persist into adult life, and resemble depressive and anxious human symptomatology.^26^ Collectively, these findings suggest that the impact of exposure to maternal depression in pregnancy and exposure to child maltreatment may be a part of the same pathway for the pathogenesis of depressive disorders. We sought to test this hypothesis by data recently collected through a new wave of assessment of offspring participants (now young adults) of the South London Child Development Study (SLCDS), a prospective longitudinal birth cohort study setup by recruiting pregnant women in 1986.^4,18,27–29^ We aimed to test the following hypotheses:

- there is an association between offspring exposure to maternal depression in pregnancy and depression in adulthood;
- child maltreatment mediates the effect of exposure to maternal depression in pregnancy on adulthood depression.

## Method

### Design

**Fig. 1:**
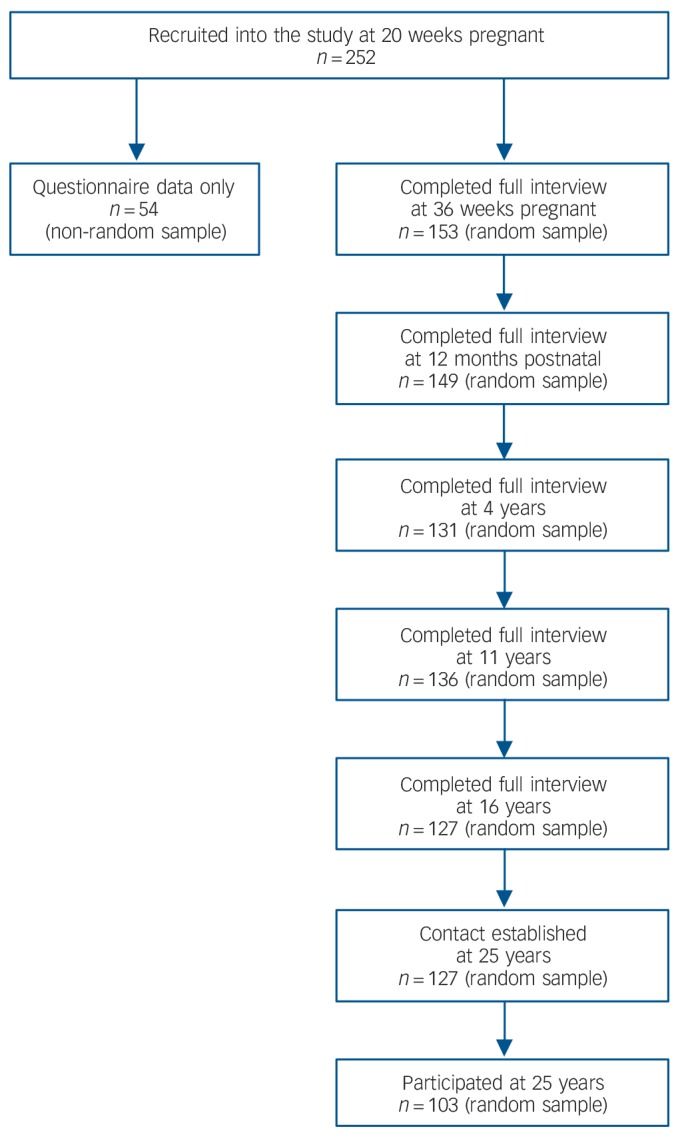
Flowchart of study participation.

The SLCDS is a prospective longitudinal UK birth cohort study that was setup in 1986.^4,18,27–29^ All pregnant women who approached either of two South London National Health Service antenatal clinics between January 1st 1986 and December 31st 1986 were invited to take part in the SLCDS. One-to-one clinical interviews were carried out with expectant women at 20 and 36 weeks of pregnancy and 3 and 12 months postpartum, and with offspring and mothers at 4, 11, 16 and 25 years. Two hundred and fifty-two women participated in the first assessment visit at 20 weeks pregnant. Because of time constraints, a 75% randomised subsample (hereon referred to as ‘the random sample’) were selected for interview at 36 weeks pregnant and 3 months postnatal, with the remaining women completing postal self-report questionnaires only. The random sample did not differ statistically in any sociodemographic or clinical characteristics in comparison with the non-random sample, and is the sample of interest in this paper.^27^ Figure 1 depicts the progress of participation for the random sample from SLCDS onset to 25 years. At each phase of the study independent researchers who were unaware of the content of previous interviews interviewed offspring and mothers.

### Sample

The sample comprised 103 offspring interviewed at age 25, and their mothers. This represents 76.3% of the offspring (*n* = 135) that were interviewed previously in childhood and adolescence. Characteristics of the sample at 25 years are presented in Table 1.

**Table 1:** Characteristics of the sample at 25 years (*n* = 103)

Characteristic	Statistic
Maternal age at index pregnancy, *M* (s.d.)	26.2 (4.8)

Maternal social class, % middle class	12.6

Maternal marital status in pregnancy, % married	63.1

Offspring age at assessment, *M* (s.d.)	25.1 (0.7)

Offspring gender, % female	52.4

Offspring ethnicity, % White British	72.8

Offspring education, % GCSEs or higher	89.3

Offspring marital status, % married	8.7

### Measures

#### Maternal depression in pregnancy

Maternal prenatal depression was assessed at 20 and 36 weeks of pregnancy by the Clinical Interview Schedule (CIS).^30^ International Classification of Diseases, Ninth Revision (ICD-9) diagnoses were made of the women's current mental state over the 2 weeks before each assessment. A dichotomous variable was created that detailed whether a mother had been clinically depressed at either time point in pregnancy (0 = non-depressed; 1 = depressed).

#### Maternal depression in the postnatal period (birth to 1 year)

At 3 and 12 months postnatal mothers were interviewed about their current mental state (past 2 weeks) by the CIS, from which ICD-9 depressive disorder diagnoses were rated. Mothers were also interviewed retrospectively at 4 years using the lifetime version of the Schedule for Affective Disorders and Schizophrenia (SADS-L) about their mental state during the entire first postnatal year, from which Research Diagnosis Criteria ratings of depression across the first postnatal year were generated.^31^ Postnatal depression was defined by the combined 3-month, 12-month and retrospective interviews; if a mother met criteria for a depressive disorder on any one of these interviews, she was rated as having experienced postnatal depression (0 = non-depressed; 1 = depressed).

#### Maternal depression during the offspring's childhood (1–16 years)

At 4, 11 and 16 years mothers were interviewed about their current mental state and experience of depression retrospective to the previous assessment using the SADS-L. Current and “retrospective to last visit” data were used to assess the occurrence of maternal depression over the child's lifetime. Variables were created to measure the children's exposure to maternal depression during early (between 1 and 4 years), middle (between 4 and 11 years) and late childhood (between 11 and 16 years). For each time interval, depression was rated if a mother met criteria for depression currently or retrospective to the last visit. We generated a ‘chronicity’ variable which summed the number of periods a mother reported being depressed (0–3 periods); moreover, a binary variable of whether the mother had ever experienced depression in the period between the child's 1st and 16th birthday was also generated (0 = non-depressed; 1 = depressed).

#### Offspring child maltreatment

Offspring exposure to child maltreatment (physical abuse, sexual abuse, emotional abuse or neglect up to age 17), was rated based on two independent assessment measures: the Childhood Experience of Care and Abuse Questionnaire (CECA.Q) conducted with the offspring at 25 years, and the Child and Adolescent Psychiatric Assessment (CAPA) conducted with offspring and primary caregiver (in most cases the mother) at 11 and 16 years.^32–34^ Physical and sexual abuse were rated based on offspring reports of severe incidents provided at 25 years by the CECA.Q (rated in accordance with cut-off guidelines published by Bifulco and colleagues),^32^ combined with the joint offspring and parental reports of severe instances of sexual and physical abuse provided at 11 and 16 years by the CAPA. For CAPA recorded incidents, physical abuse was rated if respondents reported incidents of abuse that involved at least some physical injury or force with potential for such, whilst sexual abuse was defined as incidents in which a perpetrator involved the offspring in activities for the perpetrator's own sexual gratification, such as fondling, oral contact, genital or anal intercourse. Emotional abuse and neglect were indexed through offspring ratings of severe parental antipathy and severe parental neglect up to 17 years by the CECA.Q in accordance with the rating guidelines published by Bifulco and colleagues.^32^ A binary variable of maltreatment was rated if any one of the three types of severe abuse (physical, sexual, emotional) or severe neglect were ever present (0 = non-maltreated, 1 = severe maltreatment). We also generated a continuous ‘severity’ variable that summed the number of forms of severe abuse and neglect the offspring ever experienced (0–4).

#### Offspring adulthood depression

Offspring depression (DSM-IV diagnoses of major depressive disorder (MDD), depressive disorder not otherwise specified (NOS) and dysthymic disorder) between ages 18 and 25 was assessed at 25 years by the Structured Clinical Interview for DSM-IV Axis I Disorders, Clinician Version.^35^ Diagnoses were rated in conjunction with the study psychiatrist. A binary variable indicating a diagnosis of depression was generated (0 = non-depressed, 1 = depressed). A continuous ‘severity’ variable was also constructed from the highest number of DSM-IV depressive symptoms reported in any given episode.

#### Confounding variables

The following risk variables were included as potential confounders:^36^ maternal age in pregnancy (in years); maternal ethnicity (White British (0) versus not White British (1)); maternal social class (middle class (0) versus working class (1)); maternal education (basic qualifications (O-levels) or higher (0) versus no qualifications (1)); maternal marital status in pregnancy (married (0) versus unmarried (1)); maternal psychiatric history (depression, anxiety, substance abuse, psychosis; 0 = no history, 1 = history); maternal prenatal anxiety (mean score on the anxiety subscale of the Leeds Anxiety Scale in pregnancy); maternal prenatal smoking (mean cigarettes smoked per day); maternal prenatal drinking (mean number of alcohol units drunk per week); offspring gestational age (whole weeks); offspring birth weight (grams); offspring gender (male (0) versus female (1)); offspring ethnicity (White British (0) versus not White British (1)); offspring education (at least one GCSE grade A*–C (0) versus none (1)); offspring IQ (at 16 years).

### Ethics

Full ethical approval was obtained for all stages of the study. At 25 years from London – Camberwell St Giles National Research Ethics Service Committee (reference number: 11/LO/0812).

### Data analysis

Analysis proceeded in three main steps. First, we performed univariate analyses to assess the relationships between maternal prenatal depression, offspring childhood factors (i.e. child maltreatment, maternal depression in offspring childhood), offspring adulthood depression and other risk variables. Next, we used hierarchical regression analyses to test for independent association between maternal depression in pregnancy and offspring adulthood depression. Finally, mediation analysis was performed to test whether environmental factors mediated the association between maternal depression in pregnancy and offspring adulthood depression.

All statistical analyses were conducted in IBM SPSS Statistics Version 21 (IBM Ltd., Portsmouth, UK). Log transformations were applied to improve the normality of the data. For data that did not benefit from transformation, non-parametric statistical tests were applied. Mediation analysis was conducted using PROCESS for SPSS Version 2.041.^37^ Ordinary least squares (OLS) regression was used to calculate path estimates and estimates of the size of the direct and indirect effects. Bootstrap confidence intervals were generated as an inferential statistical test of the indirect effect.^37^ In all analyses the number of bootstrap samples was set to 10 000 and 95% bias-corrected bootstrap confidence intervals (CIs) generated.

## Results

### Descriptives

Thirty-nine (37.9%) offspring met criteria for a diagnosis of DSM-IV depression in adulthood. Twenty-nine (74.4%) of the offspring with a diagnosis of depression had experienced at least one MDD episode, five (12.8%) experienced dysthymia and five (12.8%) depressive disorder NOS; the mean number of DSM-IV depressive symptoms amongst depressed offspring was 6.0, s.d. = 1.6. Thirty-five offspring (34.0%) were exposed to maternal depression in pregnancy, 20 (57.1%) of whom were further exposed in the first postnatal year. Overall, 36 (35.0%) mothers experienced postnatal depression and 64 (62.7%) mothers experienced at least one depressive episode during the offspring's childhood (1–16 years).

Thirty-six (35.0%) offspring were rated as having experienced child maltreatment. Twenty-three (22.3%) offspring experienced one form of maltreatment, three (2.9%) experienced two forms of maltreatment, six (5.8%) experienced three forms of maltreatment and four (3.9%) offspring experienced all four forms of maltreatment. Parents were observed to be perpetrators of the majority (90.0%, *n* = 18) of cases of physical abuse, but were not perpetrators of any instances of sexual abuse; stepparents, other family members, peers and non-related individuals accounted for all instances of sexual abuse. Regarding parental emotional abuse and neglect, mothers accounted for 52.9% (*n* = 9) of cases of emotional abuse and 33.3% (*n* = 5) of cases of neglect.

### Associations between maternal depression and offspring adulthood depression

As predicted by our first hypothesis, offspring exposed to maternal depression in pregnancy were 3.4 times (95% CI (1.5, 8.1), χ^2^(1) = 8.4, *P* = 0.004) more likely than those not so exposed to be depressed in adulthood. Of the 35 offspring exposed to maternal depression *in utero*, 20 (57.1%) met DSM-IV criteria for a depression diagnosis; in contrast, of the 68 non-exposed offspring, only 19 (27.9%) met criteria for a depression diagnosis. The mean number of depressive symptoms was also significantly higher amongst prenatally exposed offspring (*M* = 3.4, s.d. = 3.0) compared to non-prenatally exposed offspring (*M* = 1.7, s.d. = 2.8, *z* = −2.8, *P* = 0.004). There was a high degree of association between maternal depression in pregnancy and depression in the first postnatal year (χ^2^(1) = 11.5, *P* = 0.001, OR = 4.3, 95% CI (1.8, 10.4)). Yet, we did not find any association between maternal depression in the postnatal period and offspring depression in adulthood (χ^2^(1) = 2.1, *P* = 0.15, OR = 1.8, 95% CI (0.8, 4.2)).

However, offspring exposure to maternal depression during childhood (1–16 years) was associated significantly with offspring adulthood depression (OR = 4.2, 95% CI (1.8, 10.2), χ^2^(1) = 11.1, *P* = 0.001). Analysis suggested a ‘chronicity’ effect, whereby offspring exposed to maternal depression over a greater number of developmental periods experienced more severe depression in adulthood (*n* = 100, *r*_s_ = 0.31, *P*<0.01). The mean number of depressive symptoms amongst offspring exposed to maternal depression across 1 developmental period was 3.0 (s.d. = 3.1), amongst those exposed across two developmental periods (*M* = 3.3, s.d. = 3.5) and amongst offspring exposed across all three developmental periods (*M* = 3.6, s.d. = 3.4). Mothers depressed during the offspring's childhood were significantly more likely to have been depressed during pregnancy, 77.1%, compared to mothers not depressed during the offspring's childhood years, 22.9% (OR = 4.8, 95% CI (1.9, 12.2), χ^2^(1) = 12.0, *P*<0.001).

### Univariate associations between offspring adulthood depression and other risk factors

Next we tested for associations between offspring adulthood depression and other potential risk factors. Table 2 summarises these univariate analyses. Notably, offspring exposure to child maltreatment was associated significantly with offspring depression in adulthood (diagnoses) (OR = 2.6, 95% CI (1.1, 6.1), χ^2^(1) = 5.2, *P* = 0.022). There was a positive correlation between severity of child maltreatment and severity of adulthood depression (symptoms) (*r*_s_ = 0.30, *P*<0.01). Analysis revealed that offspring exposed to one form of maltreatment had the lowest number of depressive symptoms (*M* = 2.6, s.d. = 3.3), whilst offspring exposed to three forms experienced the greatest number of depressive symptoms (*M* = 5.5, s.d. = 3.2), indicative of a ‘dose–response’ relationship.

**Table 2:** Group differences between depressed versus non-depressed adult offspring

	Non-depressed adult offspring (*n* = 64)	Depressed adult offspring (*n* = 39)	Group effect
Maternal depression in pregnancy, %	23.4	51.3	*P* = 0.004

*Maternal characteristics*			
Age at index pregnancy, *M* (s.d.)	26.5 (4.8)	25.8 (4.9)	*P* = 0.47
Ethnicity, % White British	79.7	74.4	*P* = 0.53
Social class, % middle class	17.2	5.1	*P* = 0.12^a^
Education, % some qualifications	79.9	69.2	*P* = 0.23
Marital status in pregnancy, % married	73.4	51.3	*P* = 0.02
Previous psychiatric history, %^b^	25.4	33.3	*P* = 0.39

*Maternal perinatal factors*			
Prenatal anxiety, *M* (s.d.)	4.7 (3.1)	5.6 (3.6)	*P* = 0.25
Prenatal smoking, *M* (s.d.)	3.7 (5.8)	4.1 (6.8)	*P* = 0.90
Prenatal drinking, *M* (s.d.)	0.8 (1.5)	1.1 (2.0)	*P* = 0.19
Postnatal depression, %	29.7	43.6	*P* = 0.15

*Offspring obstetric factors*			
Birth weight, *M* (s.d.)	3399.1 (432.3)	3355.4 (582.3)	*P* = 0.66
Gestational age, *M* (s.d.)	40.1 (1.5)	39.6 (2.0)	*P* = 0.27

*Childhood factors*			
Maternal depression 1–16 years			
% exposed	40.6	74.4	*P* = 0.001
Chronicity of exposure, *M* (s.d.)^c^	0.7 (0.9)	1.3 (1.0)	*P* = 0.002

*Offspring child maltreatment*			
% exposed	26.6	48.7	*P* = 0.02
Severity, *M* (s.d.)	0.4 (0.7)	1.0 (1.3)	*P* = 0.007

*Offspring characteristics*			
Gender, % female	45.3	64.1	*P* = 0.06
Ethnicity, % White British	76.9	66.7	*P* = 0.27
Education, % some qualifications	92.2	84.6	*P* = 0.33^a^
IQ, *M* (s.d.)^d^	95.8 (16.1)	94.1 (13.8)	*P* = 0.56

Note. The independent samples *t*-test was used for group comparisons comprising continuous parametric data, whilst the Mann–Whitney test was applied to non-parametric continuous data. Pearson's chi-square test for independence was used for the analysis of categorical data.

a. Fisher's exact test applied because of one contingency table cell showing an expected cell count less than five.

b. *n* = 102 (well = 63, depressed = 39).

c. *n* = 100 (62, 38).

d. *n* = 98 (61, 37).

### Predicting offspring depression in adulthood

Hierarchical multiple logistic and linear regression models were applied to assess whether mothers' depression during pregnancy predicted their offspring's adulthood depression diagnoses and symptoms, respectively. When maternal postnatal depression and associated sociodemographic risks (i.e. maternal marital status in pregnancy, offspring gender) were entered into the models at the first step, maternal prenatal depression predicted significantly both offspring adulthood depression diagnoses and symptoms, respectively (Wald statistic 5.7, *P* = 0.02, OR = 3.1, 95% CI (1.2, 7.7), model χ^2^(3) = 12.5, *P* = 0.006; *B* = 1.6, *t* = 2.5, *P* = 0.015, 95% CI (0.3, 2.9), model *R*^2^ = 0.14, *F*(4, 98) = 4.0, *P* = 0.005). However, when childhood factors (i.e. child maltreatment, maternal depression 1 to 16 years) were entered at the second steps, prenatal maternal depression no longer predicted significantly offspring depression. We did not find any evidence of statistical moderation of maternal depression in pregnancy on offspring depression in adulthood by either child maltreatment or maternal depression during the offspring's childhood.

### Pathways from maternal depression in pregnancy to offspring adulthood depression

**Fig. 2:**
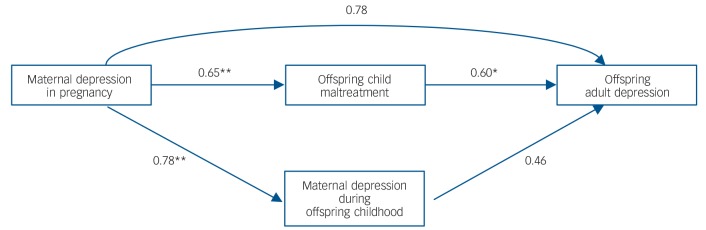
Path estimates for the multiple mediation model of the effect of maternal depression in pregnancy on offspring adulthood depression mediated by childhood risks. Note. Estimates are presented as unstandardised B coefficients. All path estimates were calculated whilst controlling for associated covariates. *P<0.05, **P<0.01.

The prevalence of child maltreatment was significantly higher amongst offspring of mothers depressed in pregnancy, 48.6% in comparison with 27.9% of offspring of non-prenatally depressed mothers (OR = 2.4, 95% CI (1.0, 5.7), χ^2^(1) = 4.3, *P* = 0.038). To investigate our second hypothesis, that offspring experience of child maltreatment mediates the association between an offspring's exposure to maternal depression in pregnancy and their experience of depression in adulthood, we conducted mediation analysis using OLS path analysis. We tested a multiple mediation model in which maternal depression in pregnancy was entered as the antecedent variable, adult offspring depressive symptoms were entered as the outcome variable, and (a) offspring child maltreatment severity and (b) maternal depression during the offspring's childhood were entered as mediator variables. Figure 2 depicts this path analytic model. In all regression models, confounding variables associated significantly with adult offspring depressive symptoms (i.e. maternal marital status in pregnancy, offspring gender) were entered as covariates.

As shown in Fig. 2, maternal depression in pregnancy predicted significantly offspring child maltreatment (*B* = 0.65, *P* = 0.002), and maternal depression during the offspring's childhood, *B*= 0.78, *P*<0.0001; moreover, offspring child maltreatment predicted significantly adult offspring depression (*B* = 0.60, *P* = 0.04). In contrast, the path between maternal depression during the offspring's childhood and offspring adulthood depression was not significant (*B* = 0.46, *P* = 0.15) indicating that the abovementioned univariate association is no longer significant when taking into account the effects of offspring child maltreatment. Table 3 summarises the regression coefficients for tests of the direct and indirect effects of offspring adulthood depression regressed on maternal depression in pregnancy. We found a significant indirect effect of maternal depression in pregnancy on offspring adulthood depression mediated by offspring child maltreatment (*B* = 0.39, 95% CI (0.04, 1.05)).

**Table 3:** Regression coefficients for the direct and indirect effects of offspring adulthood depression regressed on maternal depression in pregnancy

	Direct effect		Indirect effect			
			Offspring child maltreatment		Maternal depression in offspring childhood	
	*B* (s.e.)	95% CI	*B* (s.e.)	95% CI	*B* (s.e.)	95% CI
Maternal depression in pregnancy → offspring adulthood depression	0.78 (0.67)	−0.55, 2.11	0.39 (0.25)	**0.04, 1.05**	0.36 (0.29)	−0.09, 1.10

Note. *n* = 100. Boldface type indicates significant effect, i.e. the 95% bootstrap confidence intervals do not cross zero. Regression coefficients presented are unstandardised *B* coefficients. All path estimates were calculated whilst controlling for associated covariates. All regression models were significant at the *P*<0.01 level.

## Discussion

In the present study we used a 26-year prospective longitudinal design to demonstrate, for the first time, that exposure to maternal depression during pregnancy increases offspring vulnerability to developing clinical depression in adulthood. Moreover, we find that maternal depression in pregnancy is also associated with increased vulnerability for child maltreatment in the offspring, and that this is a mediating mechanism linking exposure to maternal depression in pregnancy and depression in early adulthood. When the SLCDS started in 1986, it was the first UK longitudinal birth cohort to investigate clinical depression in pregnancy prospectively, and thus it is the only such cohort today with data available on offspring psychopathology in adulthood.^4,18,27–29^

### Maternal depression during pregnancy and offspring adulthood depression

Our finding that offspring exposure to maternal depression during pregnancy is associated with depression in adulthood extends previous findings from the SLCDS showing that exposure to maternal depression in pregnancy predicts offspring depression at 16 years,^4^ as well as recent findings from the Avon Longitudinal Study of Parents and Children (ALSPAC) showing that exposure to maternal depression in pregnancy predicts depression at age 18.^5^ Notably, we did *not* find that exposure to maternal depression *after* birth contributes to this association. This suggests that exposure to maternal depression specifically during pregnancy represents a unique setting for the intergenerational transmission of risk for depression, which is independent from further exposure to maternal depression after birth.

This account is in line with the theoretical premise of foetal programming, which postulates that offspring exposed to an adverse intrauterine environment, especially exposure to elevated levels of maternal glucocorticoids, can result in changes in foetal brain development in regions relevant to stress reactivity, such as the hypothalamic-pituitary-adrenal (HPA) axis, inflammatory response system and amygdala.^8,10,38–42^ Indeed, HPA axis dys-regulation and inflammation are routinely observed in depressed individuals,^43–46^ and studies have shown that pregnancy *per se* is associated with increased maternal HPA axis activity and inflammation, which can be further exacerbated by the experience of depression during this time.^47–49^

### Mediating mechanisms: the influence of child maltreatment

Our second set of findings relate to the links between maternal depression during pregnancy, offspring child maltreatment, and offspring adulthood depression. We find that offspring of mothers who were depressed during pregnancy are at increased risk of experiencing maltreatment during childhood, and – crucially – that this insult is a mediator in the association between exposure to maternal depression during pregnancy and offspring adulthood depression. These results confirm and extend our previous findings that exposure to maternal depression during pregnancy predicts childhood physical and sexual abuse as well as harsh discipline, as well as findings that maternal depression and anxiety during pregnancy predict peer victimisation.^11,12,18^

The relationship between a mother experiencing depression during pregnancy and the increased vulnerability of her offspring to experience maltreatment may be explained by reduced maternal capacity for care, a poor maternal-offspring attachment relationship, the influence of exposure to aggregated environmental risks, such as maladaptive parenting and inter-parental conflict, and foetal programming of an emotionally labile offspring temperament.^12,18,19,50–52^ It is likely that these mechanisms co-occur, thereby culminating in elevated vulnerability for offspring maltreatment.

Indeed, a review summarised that maternal low mood during pregnancy was associated with lower maternal-foetal attachment.^50^ Furthermore, rates of child-mother secure attachment have been shown to be lower amongst maltreated preschool children in comparison with non-maltreated children.^51^ Harsher maladaptive parenting in conjunction with poorer maternal-offspring attachment could account for the high rates of maternal physical and emotional abuse that we observed. Moreover, the fact that the perpetrators of sexual abuse and of neglect were others than the mothers themselves could be accounted for by the impact of a compromised ability of depressed pregnant mothers to protect their young from other perpetrators both in the home and the wider environment.^19^ A more emotionally labile and reactive temperament among the offspring could contribute directly to their being more vulnerable to maltreatment as well as eliciting further detrimental parenting practices.^53^ Taken together, our findings support the notion that exposure to maternal depression during pregnancy and exposure to child maltreatment are likely part of a single pathway to adulthood depression.

### Strengths and limitations

The study has numerous strengths, such as the use of a prospective design starting in pregnancy through 26 years, the collection of data through one-to-one interviews and assessment of psychopathology at a clinically significant level. However, there are certain limitations. First, the small sample size did not allow for the analysis of more specific effects of individual risks, such as type of maltreatment. Second, the SLCDS is drawn from an urban, predominantly working class population of families of white ethnic origin. Whilst this could be deemed a high-risk population with high prevalence rates of psychopathology, a recent epidemiological study demonstrated prevalence of common mental disorders to be four-fold higher amongst urban London residents compared to residents of other UK cities,^54^ suggesting the cohort to indeed be representative of larger urban populations. Nevertheless, it is also possible that our high prevalence rates could also reflect genetic transmission of depression from parent to offspring.^55^ Finally, the majority of mothers were diagnosed with ICD-9 neurotic depression during pregnancy. As this diagnosis states that anxiety can also be present along with depressed mood, it is possible that our observed effects could be attributed to anxiety, or mixed anxiety and depression, rather than depression alone.^56^

### Implications

Our study shows that exposure to maternal depression during pregnancy increases offspring vulnerability for developing depression in adulthood. We find that child maltreatment is a putative mediating mechanism in this trajectory. These findings support the notion that exposure to maternal depression during pregnancy and exposure to child maltreatment are part of a single trajectory linking early life insults to risk for adulthood depression. By intervening during pregnancy, rates of both child maltreatment and depressive disorders in the young adults could potentially be reduced. All expectant women could be screened for depression and those identified offered prioritised access to psychological therapies – as indeed is currently recommended by the UK guidelines on perinatal mental health.^57^ Moreover, our findings will inform the current debate on the use of antidepressants during pregnancy, by highlighting the adverse consequences of *not* treating depression.^58^

## References

[1] Murray L Arteche A Fearon P Halligan S Goodyer I Cooper P Maternal postnatal depression and the development of depression in offspring up to 16 years of age. J Am Acad Child Adolesc Psychiatry 2011; 50: 460–70. 21515195 10.1016/j.jaac.2011.02.001

[2] Hammen C Brennan PA Severity, chronicity, and timing of maternal depression and risk for adolescent offspring diagnoses in a community sample. Arch Gen Psychiatry 2003; 60: 253–8. 12622658 10.1001/archpsyc.60.3.253

[3] Halligan SL Murray L Martins C Cooper PJ Maternal depression and psychiatric outcomes in adolescent offspring: a 13-year longitudinal study. J Affect Disord 2007; 97: 145–54. 16863660 10.1016/j.jad.2006.06.010

[4] Pawlby S Hay DF Sharp D Waters CS O'Keane V Antenatal depression predicts depression in adolescent offspring: prospective longitudinal community-based study. J Affect Disord 2009; 113: 236–43. 18602698 10.1016/j.jad.2008.05.018

[5] Pearson RM Evans J Kounali D Lewis G Heron J Ramchandani PG Maternal depression during pregnancy and the postnatal period: risks and possible mechanisms for offspring depression at age 18 years. JAMA Psychiatry 2013; 70: 1312–9. 24108418 10.1001/jamapsychiatry.2013.2163 PMC3930009

[6] O'Donnell KJ Glover V Barker ED O'Connor TG The persisting effect of maternal mood in pregnancy on childhood psychopathology. Dev Psychopathol 2014; 26: 393–403. 24621564 10.1017/S0954579414000029

[7] Talge NM Neal C Glover V Antenatal maternal stress and long-term effects on child neurodevelopment: how and why? J Child Psychol Psychiatry 2007; 48: 245–61. 17355398 10.1111/j.1469-7610.2006.01714.x PMC11016282

[8] Glover V Annual research review: prenatal stress and the origins of psychopathology: an evolutionary perspective. J Child Psychol Psychiatry 2011; 52: 356–67. 21250994 10.1111/j.1469-7610.2011.02371.x

[9] Charil A Laplante DP Vaillancourt C King S Prenatal stress and brain development. Brain Res Rev 2010; 65: 56–79. 20550950 10.1016/j.brainresrev.2010.06.002

[10] Barker D Fetal origins of coronary heart disease. BMJ 1995; 311: 171–4. 7613432 10.1136/bmj.311.6998.171 PMC2550226

[11] Pawlby S Hay D Sharp D Waters CS Pariante CM Antenatal depression and offspring psychopathology: the influence of childhood maltreatment. Br J Psychiatry 2011; 199: 106–12. 21727235 10.1192/bjp.bp.110.087734

[12] Lereya ST Wolke D Prenatal family adversity and maternal mental health and vulnerability to peer victimisation at school. J Child Psychol Psychiatry 2013; 54: 644–52. 23121554 10.1111/jcpp.12012

[13] O'Connor TG Heron J Golding J Beveridge M Glover V Maternal antenatal anxiety and children's behavioural/emotional problems at 4 years. Report from the Avon Longitudinal Study of Parents and Children. Br J Psychiatry 2002; 180: 502–8. 12042228 10.1192/bjp.180.6.502

[14] O'Connor TG Heron J Golding J Glover V Maternal antenatal anxiety and behavioural/emotional problems in children: a test of a programming hypothesis. J Child Psychol Psychiatry 2003; 44: 1025–36. 14531585 10.1111/1469-7610.00187

[15] Van Den Bergh BRH Mulder EJH Mennes M Glover V Antenatal maternal anxiety and stress and the neurobehavioural development of the fetus and child: links and possible mechanisms. A review. Neurosci Biobehav Rev 2005; 29: 237–58. 15811496 10.1016/j.neubiorev.2004.10.007

[16] Davis EP Glynn LM Waffarn F Sandman CA Prenatal maternal stress programs infant stress regulation. J Child Psychol Psychiatry 2011; 52: 119–29. 20854366 10.1111/j.1469-7610.2010.02314.x PMC3010449

[17] Sharp H Pickles A Meaney M Marshall K Tibu F Hill J Frequency of infant stroking reported by mothers moderates the effect of prenatal depression on infant behavioural and physiological outcomes. PLoS One 2012; 7: e45446. 23091594 10.1371/journal.pone.0045446 PMC3473033

[18] Plant DT Barker ED Waters CS Pawlby S Pariante CM Intergenerational transmission of maltreatment and psychopathology: the role of antenatal depression. Psychol Med 2013; 43: 519–28. 22694795 10.1017/S0033291712001298 PMC3558981

[19] Pariante CM Depression during pregnancy: molecular regulations of mothers' and children's behaviour. Biochem Soc Trans 2014; 42: 582–6. 24646281 10.1042/BST20130246

[20] Norman RE Byambaa M De R Butchart A Scott J Vos T The long-term health consequences of child physical abuse, emotional abuse, and neglect: a systematic review and meta-analysis. PLoS Med 2012; 9: e1001349. 23209385 10.1371/journal.pmed.1001349 PMC3507962

[21] Nanni V Uher R Danese A Childhood maltreatment predicts unfavorable course of illness and treatment outcome in depression: a meta-analysis. Am J Psychiatry 2012; 169: 141–51. 22420036 10.1176/appi.ajp.2011.11020335

[22] Widom C DuMont K Czaja SJ A prospective investigation of major depressive disorder and comorbidity in abused and neglected children grown up. Arch Gen Psychiatry 2007; 64: 49–56. 17199054 10.1001/archpsyc.64.1.49

[23] Bifulco A Moran PM Baines R Bunn A Stanford K Exploring psychological abuse in childhood: II. Association with other abuse and adult clinical depression. Bull Menninger Clin 2002; 66: 241–58. 12448629 10.1521/bumc.66.3.241.23366

[24] Green JG McLaughlin KA Berglund PA Gruber MJ Sampson NA Zaslavsky AM Childhood adversities and adult psychiatric disorders in the national comorbidity survey replication I: associations with first onset of DSM-IV disorders. Arch Gen Psychiatry 2010; 67: 113–23. 20124111 10.1001/archgenpsychiatry.2009.186 PMC2822662

[25] Wise LA Zierler S Krieger N Harlow BL Adult onset of major depressive disorder in relation to early life violent victimisation: a case-control study. Lancet 2001; 358: 881–7. 11567704 10.1016/S0140-6736(01)06072-X

[26] Nemeroff CB Neurobiological consequences of childhood trauma. J Clin Psychiatry 2004; 65: 18–28. 14728093

[27] Sharp D Childbirth Related Emotional Disorders in Primary Care: A Longitudinal Prospective Study. University of London, 1992.

[28] Sharp D Hay DF Pawlby S Schmücker G Allen H Kumar R The impact of postnatal depression on boys' intellectual development. J Child Psychol Psychiatry 1995; 36: 1315–36. 8988269 10.1111/j.1469-7610.1995.tb01666.x

[29] Hay DF Pawlby S Waters CS Perra O Sharp D Mothers' antenatal depression and their children's antisocial outcomes. Child Dev 2010; 81: 149–65. 20331659 10.1111/j.1467-8624.2009.01386.x

[30] Goldberg DP Cooper B Eastwood MR Kedward HB Shepherd M A standardized psychiatric interview for use in community surveys. Br J Prev Soc Med 1970; 24: 18–23. 5435083 10.1136/jech.24.1.18 PMC1059220

[31] Spitzer RL Endicott J Robins E Research diagnostic criteria: rationale and reliability. Arch Gen Psychiatry 1978; 35: 773–82. 655775 10.1001/archpsyc.1978.01770300115013

[32] Bifulco A Bernazzani O Moran PM Jacobs C The childhood experience of care and abuse questionnaire (CECA.Q): validation in a community series. Br J Clin Psychol 2005; 44: 563–81. 16368034 10.1348/014466505X35344

[33] Smith N Lam D Bifulco A Checkley S Childhood experience of care and abuse questionnaire (CECA.Q). Validation of a screening instrument for childhood adversity in clinical populations. Soc Psychiatry Psychiatr Epidemiol 2002; 37: 572–9. 12545234 10.1007/s00127-002-0589-9

[34] Angold A Costello EJ The child and adolescent psychiatric assessment (CAPA). J Am Acad Child Adolesc Psychiatry 2000; 39: 39–48. 10638066 10.1097/00004583-200001000-00015

[35] First MB Spitzer RL Gibbon M Williams JB Structured Clinical Interview for DSM-IV Axis I Disorders, Clinician Version (SCID-CV). American Psychiatric Press, 1996.

[36] Thapar A Rutter M Do prenatal risk factors cause psychiatric disorder? Be wary of causal claims. Br J Psychiatry 2009; 195: 100–1. 19648537 10.1192/bjp.bp.109.062828

[37] Hayes AF Introduction to Mediation, Moderation, and Conditional Process Analysis: A Regression-based Approach. The Guilford Press, 2013.

[38] Seckl JR Holmes MC Mechanisms of disease: glucocorticoids, their placental metabolism and fetal “programming” of adult pathophysiology. Nat Clin Pract Endocrinol Metab 2007; 3: 479–88. 17515892 10.1038/ncpendmet0515

[39] Reynolds RM Glucocorticoid excess and the developmental origins of disease: two decades of testing the hypothesis – 2012 Curt Richter Award Winner. Psychoneuroendocrinology 2013; 38: 1–11. 22998948 10.1016/j.psyneuen.2012.08.012

[40] Marques AH O'Connor TG Roth C Susser E Bjørke-Monsen A.-L The influence of maternal prenatal and early childhood nutrition and maternal prenatal stress on offspring immune system development and neurodevelopmental disorders. Front Neurosci 2013; 7: 120. 23914151 10.3389/fnins.2013.00120 PMC3728489

[41] Rifkin-Graboi A Bai J Chen H Hameed WB Sim LW Tint MT Prenatal maternal depression associates with microstructure of right amygdala in neonates at birth. Biol Psychiatry 2013; 74: 837–44. 23968960 10.1016/j.biopsych.2013.06.019

[42] Buss C Davis EP Shahbaba B Pruessner JC Head K Sandman CA Maternal cortisol over the course of pregnancy and subsequent child amygdala and hippocampus volumes and affective problems. Proc Natl Acad Sci USA 2012; 109: E1312–9. 22529357 10.1073/pnas.1201295109 PMC3356611

[43] Pariante C Lightman SL The HPA axis in major depression: classical theories and new developments. Trends Neurosci 2008; 31: 464–8. 18675469 10.1016/j.tins.2008.06.006

[44] Dowlati Y Herrmann N Swardfager W Liu H Sham L Reim EK A meta-analysis of cytokines in major depression. Biol Psychiatry 2010; 67: 446–57. 20015486 10.1016/j.biopsych.2009.09.033

[45] Zunszain PA Anacker C Cattaneo A Carvalho LA Pariante CM Glucocorticoids, cytokines and brain abnormalities in depression. Prog Neuropsychopharmacol Biol Psychiatry 2011; 35: 722–9. 20406665 10.1016/j.pnpbp.2010.04.011 PMC3513408

[46] Nemeroff CB Vale WW The neurobiology of depression: inroads to treatment and new drug discovery. J Clin Psychiatry 2005; 66: 5–13. 16124836

[47] O'Keane V Lightman S Marsh M Pawlby S Papadopoulos AS Taylor A Increased pituitary-adrenal activation and shortened gestation in a sample of depressed pregnant women: a pilot study. J Affect Disord 2011; 130: 300–5. 21093926 10.1016/j.jad.2010.10.004

[48] Coussons-Read ME Okun ML Nettles CD Psychosocial stress increases inflammatory markers and alters cytokine production across pregnancy. Brain Behav Immun 2007; 21: 343–50. 17029703 10.1016/j.bbi.2006.08.006

[49] Jung C Ho JT Torpy DJ Rogers A Doogue M Lewis JG A longitudinal study of plasma and urinary cortisol in pregnancy and postpartum. J Clin Endocrinol Metab 2011; 96: 1533–40. 21367926 10.1210/jc.2010-2395

[50] Alhusen JL A literature update on maternal-fetal attachment. J Obstet Gynecol Neonatal Nurs 2008; 37: 315–28. 10.1111/j.1552-6909.2008.00241.x PMC3027206 18507602

[51] Stronach EP Toth SL Rogosch F Oshri A Manly JT Cicchetti D Child maltreatment, attachment security, and internal representations of mother and mother-child relationships. Child Maltreat 2011; 16: 137–45. 21339198 10.1177/1077559511398294

[52] Feldman R Weller A Zagoory-Sharon O Levine A Evidence for a neuroendocrinological foundation of human affiliation: plasma oxytocin levels across pregnancy and the postpartum period predict mother-infant bonding. Psychol Sci 2007; 18: 965–70. 17958710 10.1111/j.1467-9280.2007.02010.x

[53] Ge X Conger RD Cadoret RJ Neiderhiser JM Yates W Troughton E The developmental interface between nature and nurture: a mutual influence model of child antisocial behavior and parent behaviors. Dev Psychol 1996; 32: 574–89.

[54] Hatch SL Woodhead C Frissa S Fear NT Verdecchia M Stewart R Importance of thinking locally for mental health: data from cross-sectional surveys representing South East London and England. PLoS One 2012; 7: e48012. 23251330 10.1371/journal.pone.0048012 PMC3520993

[55] Silberg JL Maes H Eaves LJ Genetic and environmental influences on the transmission of parental depression to children's depression and conduct disturbance: an extended Children of Twins study. J Child Psychol Psychiatry 2010; 51: 734–44. 20163497 10.1111/j.1469-7610.2010.02205.x PMC2891390

[56] World Health Organization Mental Disorders: Glossary and Guide to Their Classification in Accordance with the 9th Revision. World Health Organization Press, 1978.

[57] National Collaborating Centre for Mental Health Antenatal and Postnatal Mental Health: Clinical Management and Service Guidance. NICE Clinical Guidelines, No. 45. The British Psychological Society, 2007. 21678630

[58] Ross LE Grigoriadis S Mamisashvili L Vonderporten EH Roerecke M Rehm J Selected pregnancy and delivery outcomes after exposure to antidepressant medication: a systematic review and meta-analysis. JAMA Psychiatry 2013; 70: 436–43. 23446732 10.1001/jamapsychiatry.2013.684

